# Impact of coping profiles on social identity in a youth chronic pain recommender system: qualitative descriptive study

**DOI:** 10.3389/fdgth.2026.1727940

**Published:** 2026-09-15

**Authors:** Antonia Barbaric, Joseph Saliba, Anya Nair, Vina Mohabir, Jennifer Stinson, Alex Mariakakis, Quynh Pham, Joseph A. Cafazzo, Chitra Lalloo

**Affiliations:** 1 Institute of Health Policy, Management, and Evaluation, University of Toronto, Toronto, ON, Canada; 2 Centre for Digital Therapeutics, University Health Network, Toronto, ON, Canada; 3 Child Health Evaluative Sciences, Research Institute, The Hospital for Sick Children, Toronto, ON, Canada; 4 Lawrence Blomberg Faculty of Nursing, University of Toronto, Toronto, ON, Canada; 5 Department of Computer Science, University of Toronto, Toronto, ON, Canada; 6 Telfer School of Management, University of Ottawa, Ottawa, ON, Canada

**Keywords:** digital health, health recommender system, pain coping, pediatric chronic pain, self-management, social identity

## Abstract

**Background:**

Chronic pain affects one in five children and adolescents worldwide yet remains one of the most underrecognized and undertreated illnesses, emphasizing the need for accessible, personalized digital health support. The Power over Pain portal delivers such support through a virtual stepped-care pathway, but like many digital interventions, is vulnerable to declining user engagement over time. Health recommender systems (HRSs) can personalize resource delivery and combat “choice overload,” thereby encouraging sustained use. This study investigates the application of a health recommender system (HRS) as a way of increasing engagement with relevant virtual care resources for pediatric chronic pain. The perceived value of the HRS was evaluated through a qualitative descriptive study involving ten participants diagnosed with chronic pain.

**Results:**

Thematic analysis revealed four main themes: (1) HRS serves as a catalyst for important dialogue in routine care; (2) coping results from the HRS are selectively applied by users according to their needs; (3) the HRS creates a personable experience, positively influencing willingness to engage in care; (4) interacting with the HRS may affirm participants’ social identities, fostering a sense of belonging in the pain community and potentially enhancing long-term resource engagement.

**Conclusion:**

These findings challenge the traditional focus on recommendation accuracy alone, suggesting instead that the effectiveness of an HRS may also be influenced by design features that support validation, shared experience, and potential social identification, which can deepen engagement and promote involvement in care. Future work should investigate the impact of mechanisms that reinforce users’ social identities to understand their effects on sustained use and self-management behaviors.

## Background

### Introduction

Chronic pain is defined as pain that lasts longer than three months and is associated with unpleasant sensory and emotional experiences (1, 2). It is one of the most misunderstood, under-recognized, and under-treated illnesses globally, especially in the pediatric space (1, 3–7). The use of virtual care is a treatment approach meant to provide a continuum of support for patients by allowing them to remotely connect with relevant care (8, 9), alleviating some of the challenges with accessing in-person approaches (10–12). The Power over Pain (PoP) portal (13–18) is a virtual stepped care (19) platform for youth (ages 12–18) to manage their chronic pain (20). The portal was created during the heights of the COVID−19 pandemic in response to the growing need for accessible evidence-based virtual resources for Canadian youth living with chronic pain. Currently, it captures data about a user based on their responses to five pain interference self-assessment questionnaires (21–26), and provides recommendations and access to a suite of relevant, evidence-informed virtual care resources (18, 20). Consistent engagement with the platform and its resources remains an ongoing challenge, as commonly observed with digital health applications (27). Furthermore, the effectiveness of these digital intervention is solely dependent on how frequently and meaningfully users choose to interact with the therapy, thereby directly influencing the benefits they experience in managing their chronic pain. Engagement with therapeutics is especially relevant in a chronic disease context, where treatment is primarily focused on long-term symptom management rather than cures.

### Related works

Health recommender systems (HRSs) provide recommendations tailored to an individual based on their needs, preferences, and, in this study, their health status. The overall goal is to share relevant resources to encourage more consistent user engagement with interventions and/or behaviors appropriate to their health needs. The PoP portal lends itself well to the application of an HRS because, as a digital intervention, it is vulnerable to low user engagement (27). The multitude of resources available may leave users feeling overwhelmed, a phenomenon often referred to as the “paradox of choice” (28). Most HRSs employ machine learning (ML) to implement recommendation techniques, such as content-based filtering, collaborative filtering, and hybrid methods that combine the two, each offering a different approach to generating recommendations through information retrieval and processing (29). To date, multiple reviews have been published about HRSs in health applications (29–38), including one of our earlier works focused on the use of HRS for chronic disease management (39). Work by Cheung et al. (40) presents “Intellicare Hub”, an HRS that provides access to a suite of digital apps for mental health management. Among existing literature, this is the most closely related to our work, as it similarly uses an HRS to enhance user engagement by simplifying access to relevant digital health resources. Now, more recent research has begun exploring generative artificial intelligence (AI) as a novel approach to generating recommendations through the use of large language models (LLMs) (41, 42). Health-based LLM recommender systems share a similar purpose with traditional HRSs that range from lifestyle guidance, such as diet (43), to more clinically focused areas like medication adherence (44), treatment decision making (45), and diagnosis (46). This growing body of research emphasizes the value of personalized recommendation systems as a means of supporting sustained engagement with digital therapeutics.

### HRS version of the PoP portal

For the purposes of this study, a standalone HRS-based PoP portal was developed under the guidance of a pragmatic, user-centered design approach (47) and supplemented with HRS methods from Calero Valdez et al. (37) Notable changes from the current version include (1) an additional 10-item questionnaire meant to capture more data about a user's pain coping style [see Supplementary file 1, Supplementary Table 1], and (2) a new algorithm consisting of HRS techniques to generate resource recommendations for the user. Existing questionnaires in the PoP portal focused only on pain ratings, so we created an additional questionnaire that captures how individuals manage and respond to pain using four main profiles and descriptions from Claar et al.'s work: (1) Avoidant: catastrophize and disengage from activities, (2) Dependent: catastrophize, but seek social support, (3) Engaged: utilize problem-solving strategies and seek social support, and (4) Self-Reliant: utilize accommodative coping strategies. The first set of results represent how similar the user's responses are to each of the four profiles [see example Supplementary file 1, Supplementary Figure 1). To generate the scores, a content-based technique is used by implementing cosine similarity (48) to calculate similarity scores between questionnaire responses and each coping profile according to 10 predefined coping tags outlined by Claar et al. (49) Each questionnaire response option was represented by a binary vector across these 10 coping tags: problem solving, seeking social support, rest, self-isolation from others, disengagement from activities, catastrophizing thoughts, self-encouragement, distracting or ignoring, acceptance thoughts, and minimizing. The vectors corresponding to a user's selected responses were averaged to generate a user-level coping vector, which was then compared with the predefined vector for each of the four coping profiles using cosine similarity.

The second set of results is the resource recommendations that are generated after the user fills out the pain interference questions currently on the existing PoP portal [see example in Supplementary file 1, Supplementary Figure 2]. These results are generated using a hybrid-based HRS approach: first, a content-based TF-IDF (Term Frequency-Inverse Document Frequency) vectorizer algorithm (50) is used to match each pain resource to each coping profile, which is multiplied by a weighted factor of the user's profile results. TF-IDF was selected as a simple, unsupervised method that required no labelled training data and was appropriate for matching the small, structured corpus used in this proof-of-concept system. Then, a collaborative-based approach is employed by using data collected from the Abstract platform (51) [formally known as Analytics Platform to Evaluate Effective Engagement (APEEE)]. The data were preprocessed by excluding test accounts and users with incomplete questionnaire or resource rating data. Users were mapped in a vector space based on their pain interference questionnaire scores for sleep, mood, worry, pain, and substance use. A k-nearest neighbour algorithm (52) with Euclidean distance approach was used to identify the five users with the most similar questionnaire scores. Several values of k were explored during development, and k = 5 was selected pragmatically as appropriate for the small proof-of-concept dataset; no formal sensitivity analysis or hyperparameter optimization was performed. For these neighbouring users, engagement with each resource was retrieved based on whether they selected “Learn More,” “Like,” or “Completed.” These actions were assigned increasing weights, with “Completed” receiving the highest weight and “Learn More” the lowest. The weighted engagement values were aggregated across the five neighbours to generate a score from 0 to 1 for each resource, with higher values indicating greater potential relevance to the target user.

A 75%/25% weighting of content-based and collaborative-based results, respectively, was applied to the list of resources. This weighting was selected pragmatically for this proof-of-concept prototype rather than through formal optimization. Greater weight was assigned to the content-based component because the collaborative-filtering component relied on a limited volume of prior user-engagement data, whereas the content-based component could generate recommendations directly from the user's coping-profile results. To support easier navigation and reduce the number of options presented, the weighted recommendations were then combined with the recommendations from the current portal to identify and display the two highest-scoring resources from each resource category, with future iterations potentially allowing users to expand the results to view additional similarly ranked resources.

### Study aim

Building on the previously described HRS-enabled PoP portal, this study's focus was to evaluate the added value that its recommender-based approach brings to the user experience by investigating the following research question: What is the perceived value of incorporating HRS-based recommendations in helping youth manage their chronic pain?

## Methods

### Overview

A qualitative descriptive study using thematic analysis (53–55) was performed to understand participants' experiences interacting with the HRS and to assess its relevance for pain management. This research method stays close to the data by “provid[ing] straightforward descriptions of experiences” (56, 57) and is appropriate when there is little known about a topic (57). “Perceived value” in this study was assessed through five key dimensions: (1) framing, which considers how supportive and engaging the presentation of questions and results is; (2) clarity, focusing on the ease of understanding the information; (3) informativeness, examining the sufficiency of information provided for interpretation; (4) applicability, assessing the relevance and personal meaning of the content; and (5) usefulness, determining how beneficial the questionnaire and results are in guiding decisions and actions.

Each session was led by the study coordinator (AB) in the presence of another researcher (AN). Sessions included participant interaction with the prototype followed by discussions guided by a semi-structured interview protocol [see Supplementary file 2]. Interviews were conducted between July 2024 and February 2025 online via Zoom, each lasting 30 to 45 min. Study details are presented in accordance with the consolidated criteria for reporting qualitative research (COREQ) guidelines (58) [see Supplementary file 3].

### Ethical considerations

Ethics approval was obtained from the Hospital for Sick Children (CTO ID: 4025) and the University of Toronto Research Ethics Boards (Protocol ID #46965).

### Participant selection and characteristics

Six participants from an existing PoP study and four patient partners were recruited using convenience sampling (59). Eligibility criteria for the participants included being part of the existing study and having adequate proficiency in English. Patient partners were not required to have prior familiarity with the PoP portal and needed to have adequate proficiency in English. Participants were recruited by the Sickkids study coordinator (AN) and provided written informed consent.

All participants (*N* = 10) were familiar with the PoP portal. Participants ranged in age from 14 to 28 years (mean 17.9, SD 4.5). Participants self-identified their gender as women (*n* = 7, 70%), men (*n* = 2, 20%), or preferred not to disclose (*n* = 1, 10%). Participants self-identified their race as follows: Black (*n* = 1, 10%), Latin American (*n* = 1, 10%), Multiracial: White and Chinese (*n* = 1, 10%), South Asian (*n* = 1, 10%), and White (*n* = 6, 60%). Duration of chronic pain varied: < 6 months (*n* = 1, 10 %), 6 months – < 1 year (*n* = 2, 20 %), 1 – 3 years (*n* = 3, 30 %), and > 3 years (*n* = 4, 40 %).

Although the PoP portal is intended for youth aged 12–18 years, patient partners older than 18 years were included because of their lived experience of pediatric chronic pain and their role in providing retrospective feedback on the proposed features.

### Data collection and analysis

Data saturation was considered achieved when iterative review of the interview transcripts and field notes no longer identified substantively new codes or themes relevant to the study aim. Triangulation of these data sources was used to strengthen the credibility of the analysis. An interview guide was co-developed with Sickkids pain team based on the study aim and organized into four areas of inquiry: questionnaire content, coping-profile results, recommended resources, and overall impressions of the proposed feature. Questions across these sections were designed to examine the five dimensions of perceived value: framing, clarity, informativeness, applicability, and usefulness, while allowing the interviewer to adopt the discussion based on participants' responses and engagement. Mapping of representative interview questions to each construct is provided in Supplementary file 2, Supplementary Table 1.

Interviews were audio-recorded, transcribed, and analyzed using NVivo software (Version 15). Interview transcripts and field notes were independently reviewed by two researchers (AB and JS), who used analytical memoing to become familiar with the data. The researchers then met to compare their observations and align on the application of the predefined deductive codes based on the five constructs: framing, clarity, informativeness, applicability, and usefulness. One transcript was coded collaboratively using both deductive and inductive approaches to establish a shared interpretation of the coding framework. The remaining transcripts were coded independently, with intermittent meetings to compare findings, discuss emerging codes, refine the codebook as needed, and resolve differences through consensus. No formal inter-coder reliability statistic was calculated, as coding agreement was established through this iterative consensus process. A second coding cycle was then used to reorganize and prioritize codes into subcategories, categories, and overarching themes.

## Results

The themes presented in this section have been intentionally organized to build upon one another, enhancing the overall understanding of insights and their implications (55). The most applicable dimensions from the perceived value definition outlined earlier have also been identified for each theme in the titles.

### Participants appreciated the additional questionnaire and emphasized the need to ask these questions as part of chronic pain care (framing; informativeness; applicability)

The additional coping questionnaire stood out as valuable and essential to participants, despite already needing to fill out numerous forms. As Participant 04 noted, “*It would just add so much more depth* [to the portal].” Questions that stood out to participants were either because they were rarely asked about the topics or because they thought it was information worth sharing with their care team:

“I do like this question because, like, that's a big thing that the other portal didn't have—it didn't have anything about how you cope with it…Because a lot of people don't actually ask that.”—Participant 06

Users' willingness to disclose additional information about themselves is not an expectation but rather an indication that they trust and see value in sharing this information (60). Guiding methods for HRS design (37, 61) emphasize the need to prioritize transparency and explanation of results, which was effective based on Participant 03's feedback: “*I really trust this*.” The explanations were also critical to avoid potential misinterpretation of results:

“At first, I was kind of confused on seeing the words (names for the profiles) because, typically, those words have been used about pain, when they say dependent, that's usually dependent on like some kind of substance. That's how typically I have heard it been used…but it makes more sense reading through the descriptions.”—Participant 06

The questionnaire was effective at balancing required effort and value; despite requiring low effort, participants felt that it still required reflection and was worthwhile to complete. Participant 03 described that going through the questions and thinking about how to answer them “*would be a realization*” about their health management habits, showcasing that it is an immersive and reflective experience.

This feedback confirms the added value of the questionnaire given its focus on aspects of chronic pain that are not currently captured in other care interactions. Considering their lived experiences, participants felt it was a reflective and useful tool that required appropriate effort to complete.

### Participants were able to interpret and use the results from the questionnaire as needed for their own benefit (applicability; usefulness)

Recognizing the potential risk of “diagnostic labeling” (62) with the profile results, we explored how they were interpreted by participants. Most feedback articulated strong agreement with their coping profile results, as described by Participant 01:

“I think it reflects pretty well, because even as I said, I'm not surprised that it's avoidant that is the highest.”—Participant 01

Conversely, we also noticed participants’ ability to identify and call out inaccuracies, demonstrating confidence and strong self-awareness:

“I think the engaged is accurate. I would probably put the avoidant and self-reliant profiles as a lot higher and dependent probably a little lower.”—Participant 02

The results were informative and did not impose labels on the user, with Participant 03 stating: “*It doesn't define me, but it's kind of “me” in there.*” Participant 06 describes a similar sense of control since they felt more ownership over their preferred coping style:

“It's useful to kind of think about your own pain without it kind of being pushed that “this is the right way to manage it” or “this is what you should be doing” … It's helpful to be more in control of what your kind of management style is.”—Participant 06.

Participant 04 appreciated seeing all of the profiles as part of the distribution of their results for a couple of reasons. First, it offered them validation that their emotional responses to pain are legitimate: “*Like it's still like an OK thing to feel. It's just a bigger chunk of what I'm feeling, compared to the other parts.*” Second, it made them feel more informed about their pain: “*…the explanation to each kind of different “quadrant”, it makes me understand more of that separate little part of how I end up feeling.*” Patient partners articulated that the results can help them express their pain management and is similar to developing a common language.

### The additional questionnaire and results create a personable experience for the user, which could motivate them to engage with the PoP portal and become more involved in managing their health (applicability)

Curiosity was one of the main feelings expressed by participants when asked how the results made them feel:

“I'd say probably curious if anything because I've never seen these kinds of [profiles], again because I think that's because not many people ask how you're coping. So, I think that was really interesting.”—Participant 06

Feeling interested in the results creates a more interactive experience for the user, which can lead to better engagement with the portal and chronic pain more broadly, as articulated by Participant 02:

“I think the more interactive and person-based it is, the more motivating you would be to visit the portal and look through everything… I would look into the definitions of them more and probably figure out how this might help me find ways of coping with my pain. For example, here it says that I'm an engaged coper, so I might figure out what techniques are best for those kinds of people.”—Participant 02

The transparency of results through the explanations created a personable experience because participants understood how their responses were integrated. Participant 02 commented, “*Knowing that your answers were taken into consideration [is] helpful and empowering*.” This sentiment was echoed by other participants who described feelings such as reassurance (Participant 05: “*it would make me feel more secure and able to know what would be best for me.”*), comfort (Participant 01: “*I think all of them have their own way of being comforting.*”), and even pride (Participant 03: “*I'm proud to find a profile that represents me… I feel like it's me.*”).

The novelty of the questionnaire made participants feel curious about their results, offering a self-reflective experience that felt both engaging and personally relevant. Participants also described the experience of filling out the questionnaire to be personable due to the reflection it prompted and the perceived accuracy of their results. As pain experiences and coping responses vary over time, revisiting the question may continue to offer value across multiple uses.

### Interacting with the additional questionnaire leads users to action, is therapeutic, and builds a sense of community (framing; usefulness)

Feedback from participants demonstrated that the questionnaire creates actionable next steps for PoP portal users in finding relevant resources:

“For me, it would be really useful because seeing what kind of profile I have could help me find the best type of resources…So yeah, having this and being like, “Oh, well, avoidant”, so I'm gonna go like, “Ohh I respond best to self-paced and interactive models, so I can search more rapidly to find resources that help me.””—Participant 03

Meetings with patient partners highlighted a similar interest in connecting profiles to resources. From these discussions, it was suggested that including specific resource examples, in addition to a description of resource types, could be more actionable for the user. Besides the questionnaire being actionable and self-reflective, it also allowed participants to better understand what others diagnosed with chronic pain may be experiencing, which can help build a sense of community:

“I mean, like, it's interesting to see the different sections of the profiles. Just to like, know that, like, different kinds of people have different things that they're that they used to cope with pain.”—Participant 05

Participant 02 emphasized that even if the results do not fully resonate with an individual, they can at least inform individuals of different coping styles, allowing them to explore any of the aspects they find most relevant:

“And like, even if it didn't necessarily match up with what people thought they were, that might still give them ideas like ohh, it thinks I'm this, and then they might go off and do more research about whatever. I think no matter what, it would still be beneficial to see.”—Participant 02

Another therapeutic benefit of the questionnaire was articulated by Participant 04, who spoke to the reflective nature of this tool by stating that they appreciated “*getting to put like my information out there, so I'm not like bottling it up.*” Patient partner discussions described how viewing the results could make an individual's experiences with chronic pain feel more real. Similar to developing a common language, the results could help individuals think, process, and manage their pain.

## Discussion

### Preliminary findings

These study findings are the first step in evaluating the HRS version of a digital health platform by focusing on the additional patient-facing questionnaire component. This work built on the research by Claar et al. (49) and aimed to better involve individuals diagnosed with chronic pain in managing their health, thereby fostering a more collaborative approach to care.

The study participants described the HRS as a beneficial tool in helping them identify relevant resources, an anticipated study finding given the goal of this work. However, we also discovered additional, unanticipated value in the HRS: it may provide a means for individuals to discover and reflect on their social identity, which can lead to greater engagement in their health. This suggests that a recommender system's impact can extend beyond tailored recommendations: its design may implicitly employ principles of social identity approach (SIA) (63), offering a personable experience that helps users make sense of their lived experience in relation to others. Specifically, the algorithmic output presents users with their relative similarity to four coping profiles, providing categories and descriptions through which they can interpret their own coping patterns and recognize shared experiences with others living with chronic pain. Unlike most recommender systems research, participants did not describe perceived accuracy (64) as the sole determinant of the HRS's value. While it did contribute to the tool's perceived value, we found that either participants agreed with the results or had slightly different opinions but did not feel that the results were “labeling” their health status (62). These findings suggest that recommender-system feedback may provide value beyond perceived accuracy by validating users' experiences and potentially supporting social identification through mechanisms consistent with SIA. The reflective nature of the questionnaire and the expressed interest from participants in learning more about the various coping styles that exist helped foster a sense of togetherness amongst pediatric patients experiencing chronic pain (65). Using the categorization and terminology from the recommendations framed a way of thinking and provided a common language that can enable individuals to become more involved, interested, and confident in managing their health. Other questionnaire attributes also helped reinforce these observed effects, including (1) the positive framing of questions and results, (2) the inclusivity of response options, (3) the appropriate question types to allow for reflective user articulation, and (4) the questions being accessible but also easy-to-answer and reflective.

Showcasing the results as a combination of the four profiles implicitly demonstrates that there are others going through similar experiences and was beneficial to share with individuals as it can create stronger “social connection” and “enhance group formation” among the pain community (66) as described by Bastian et al. Their research (66) explored the “positive consequences” of pain through the lens of a biopsychosocial framework and found that “shared experiences of pain promote shared identity and a sense of belonging” (66). The theories of self-categorization (67) (SCT) and social identity (68) (SIT) support the categorization of profiles as a way to build a sense of community. SCT helps individuals make sense of the world by grouping themselves and others into meaningful social categories (69). In this case, the algorithmically generated coping-profile results provided a framework through which individuals could better understand their own experiences, categorize their coping patterns, and recognize commonalities with others experiencing similar struggles. As described by patient partners, the profiles and their explanations can also serve as a common language to make sense of and explain their chronic pain to others. SIT is the other component that builds on SCT which articulates how being part of a group shapes an individual's perception of self identity (63) and further builds community.

We found limited research investigating the influence of social identity in chronic disease management. Research by Grünenwald et al. (70) and Cameron et al. (68) aimed to advance the biopsychosocial model by examining social identification as a key mechanism in explaining coping behavior for individuals diagnosed with chronic pain. Their findings showcased that a strong social identification is connected to more frequent use of coping strategies and recommended creating interventions that enhance an individual's connection to the chronic pain community. Building on their findings and our own, we believe that the HRS can be beneficial to an individual in ways beyond providing personalized recommendations, specifically by validating users' experiences through the coping-profile results, which may support social identification and greater engagement in care. These proposed effects require direct evaluation in future studies.

### Study limitations

This study has two main limitations. First, the interview findings reflect a small, pre-existing user group with limited demographic diversity, which may not capture the experiences of the broader pediatric chronic pain population. Convenience sampling may also have favoured participants who were more familiar with research participation, more engaged in their care, or more interested in digital health tools. This may have introduced selection bias and limited perspectives from less engaged users. The inclusion of young adult patient partners may also have introduced perspectives that differ from those of current adolescent users, particularly because age-related differences in autonomy, health-management experience, and engagement with digital health interventions may influence how the HRS is perceived. Participants may also have provided more favourable feedback because of social desirability or a desire to be supportive of the proposed feature, despite being encouraged to provide open and honest feedback. Second, this study represents an initial proof-of-concept evaluation of the HRS version and focused primarily on the added questionnaire rather than the performance of the recommendation algorithm. Accordingly, several design decisions, including the weighting of the content-based and collaborative-filtering components, which were selected pragmatically and were not empirically optimized. The questionnaire and associated scoring approach may also be affected by differences in how users interpret the questions, select among the available response options, and use the numerical response scales, which could influence the resulting coping-profile similarity scores. Also, social identity was not directly assessed using a validated measure; its proposed role was theoretically inferred from participants' qualitative descriptions of validation, shared experience, and community. Further research is needed to validate and refine both the questionnaire and recommendation algorithm, assess the HRS's overall effectiveness in promoting uptake of evidence-based resources, and explore any additional effects, such as its impact on social identity.

## Conclusions

This research focused on a preliminary evaluation of a health recommender system (HRS) version of a digital health platform, with the aim of creating a more personalized health management experience and increasing patient involvement. Findings revealed that this version provided unexpected value. Notably, participants' accounts suggested that the additional questionnaire may support reflect, perceived social connection, and a sense of community. Future research should directly evaluate whether these experiences translate into stronger social identification and sustained engagement with care. Given the challenge of low adherence to digital health tools, the use of social identity mechanisms could be a promising avenue for improving engagement with care.

## Data Availability

The raw data supporting the conclusions of this article will be made available by the authors, without undue reservation.
