# Supplementary material for: Impact of coping profiles on social identity in a youth chronic pain recommender system: qualitative descriptive study
Source: Front Digit Health. 2026 Sep 15;8:1727940. doi: 10.3389/fdgth.2026.1727940 (PMC13619948; doi:10.3389/fdgth.2026.1727940)
Supplement: Supplementary file 1 [file Supplementaryfile1.docx]

# Supplementary file 1: HRS Coping Questionnaire

*Table 1: 10-item questionnaire with close-ended style responses, based on research published by Claar et al.* [*(Claar et al. 2008)*](https://paperpile.com/c/2aIKvw/qOyd)

| **Question #** | **Question** | **Response Options** |
| --- | --- | --- |
| 1 | When you first start feeling pain, how do you typically deal with it? | - Reach out to a family member or friend to let them know how I’m feeling - Hang out with my friends and family so that I distract myself from the pain - Hang out by myself and find things to do to distract myself from the pain - Cancel my plans and rest - Wait for the pain to pass, oftentimes feeling worried and/or anxious - Look for resources (like: medications, speaking with my doctor, or things online) to help me manage my pain |
| 2 | What is your attitude like when you're in pain? | - A positive mood, I tell myself that I will get through this - I feel motivated to find a solution to help me manage my pain - I feel worried, anxious, frustrated, sad - I downplay the pain and don't let it bother me |
| 3 | Does your pain impact other parts of your life, such as going to school, doing hobbies, or hanging out with friends? | Likert scale from 1 (No, not really) to 10 (Yes, most of the time) |
| 4 | When you're in pain, how often do you deal with it by using distractions (like focusing on other stuff besides the pain, as a way to forget about it)? | Likert scale from 1 (Never) to 10 (All the time) |
| 5 | Do you have any interest in tracking your progress on pain management in the Power Over Pain (PoP) portal? | Likert scale from 1 (No interest) to 10 (Very interested) |
| 6 | [If you reach out to others for help when you are in pain] ... who do you typically speak with? | - Family members - Friends - My care team at the hospital - People I met online, who are going through similar things as I am - I don't reach out to others (manage best by dealing with it alone) |
| 7 | [If you reach out to others for help when you are in pain] ... how do you prefer to talk to them? | - In-person - Online, video sessions - Online, chat messaging sessions - Online, community portal - I don't reach out to others (manage best by dealing with it alone) |
| 8 | Does your pain improve when you rest and limit your activities? | Likert scale from 1 (No, not really) to 10 (Yes, most of the time) |
| 9 | What kinds of resources do you prefer to use to help you learn and/or manage your pain? | - Reading articles online - Watching videos - Listening to podcasts - Interactive sessions with relevant content (like an online course) - Interactive online sessions with other people (like online chats or video calls) - Medications - I don't look for resources and instead manage in other ways |
| 10 | How much time do you think you would spend on the PoP portal to access pain management resources? | - Multiple (3+) times per week - A few (1-2) times per week - One time every few (1-2) weeks - One time every 3-4 weeks |

*Figure 1a: Example output of profile results which include a similarity score for each of the four profiles.*

*Figure 1b: Example of explanations presented to the user related to their results. Each profile explanation addresses (1) common behaviors associated with the profile, (2) typical responses from the questionnaire that the type of profile most relates to, and (3) the most suitable types of coping resources.*

*Figure 2: Resource recommendations, set-up similar to the existing PoP portal. The resource recommendations are presented in mostly the same way as the current PoP portal except for two changes: (1) an additional section for each resource explaining why it is being recommended to user57, and (2) a section highlighting feedback from other users who already used the resource, as a way of helping them identify which resource they may want to try next.*
