# Supplementary material for: Impact of coping profiles on social identity in a youth chronic pain recommender system: qualitative descriptive study
Source: Front Digit Health. 2026 Sep 15;8:1727940. doi: 10.3389/fdgth.2026.1727940 (PMC13619948; doi:10.3389/fdgth.2026.1727940)
Supplement: Supplementary file 2 [file Supplementaryfile2.docx]

# Supplementary file 2: Semi-structured interview guide

**Introduction**

*Welcome, and thank you for expressing interest to participate in this interview about potential, future PoP portal features. We are looking for your open and honest feedback, including things you liked and did not like about the design. There are no wrong answers. Please let us know if you have questions at any time, and if you need a break or would like to stop the interview just let us know. You do not have to answer any questions that you do not want to. This part of the interview session will also be audio-recorded and is required for this study. If you do not want to be audio-recorded, you cannot participate in the study. Also, as we go through the interview, we will be taking notes to be able to review at a later time. Lastly, the interview will take approximately 30 - 45 minutes, depending on your answers and the flow of the conversation. If you have a hard stop on timing please let me know and we will prioritize finishing within that time frame.*

**Potential, Future Feature #1**

*One future feature that we are thinking of implementing is a 10-item questionnaire that the user fills out after they create an account for the portal. T****his questionnaire asks about how you manage and think about your pain, currently.*** *We ask these questions to figure out how much they “match up” to different pain coping profiles that have been already figured out by previous researchers. We want to use these profiles to make more personalized resource recommendations.*

Questions about the Feature #1:

1. Are all the questions relevant to helping you find the best approach in managing your chronic pain?
   a. *Follow up:* *Were you able to answer all the questions? If not, why?* *Do the questions need to be more specific?*
   b. *Follow up: which questions are the most important to you?*
   c. *Follow up: which questions are the least important to you?*
2. Are the questions written in a way that is easy for you to understand?
3. On a scale of 1 to 10, how much effort did it take to fill out the questionnaire?
   a. *Scale:* *1 -- hardly any effort and easy to answer; 5 -- some effort and thinking required to answer; 10 -- maximum effort and difficult to choose an answer*
4. Did these questions bring about any feelings?
   a. *For example, did they make you feel: discouraged, or curious, or motivated, or sad, or even confused?*
5. (or) Did these questions make you think about anything specifically?
   a. *For example, did it make you think about anything specific related to your pain management journey?*
6. Which questions to you are a) the most important and b) the least important. Why?

*Optional (if time permits):*

1. How often would you want to fill out this questionnaire?
   a. *Probe: Are your answers to these questions something that would change often or not? For example, would your answers to these questions vary by day or every few weeks or longer? Explain why.*
2. Do you have any suggestions on how to make users feel more motivated to fill out this questionnaire?

**Potential, Future Feature #2**

*A second, future feature we might implement is a “Results” page for the 10-item questionnaire. This page would show “how much” of each pain coping profile your answers reflect, along with descriptions about the pain coping profiles, and a FAQ page.*

Questions about the “Results for Pain Coping Profiles” page on the PoP portal:

1. Do you feel that the results on this page are applicable to you and accurate in how you are feeling? OR How do the results from the questionnaire (coping profile results) compare to how you cope with pain?
   a. *Follow up:* Are they similar? If yes, in what way?
   b. *Follow up:* Are they different? If yes, in what way?
   c. *Follow up:* Is there anything about the results that you find confusing?
   d. *Follow up:* Is there anything about the results that surprised you?
   e. *Follow up:* Would you say that these results are an accurate depiction of how you choose to manage pain? I.e., are these results applicable to you?
2. [Despite whether or not they are applicable …] Do you feel that this feature is useful for you? Why or why not? If yes - what benefit do you think it will provide you and/or others?
3. Would this questionnaire help you to pick resources from the PoP portal? Why or why not? How do you currently figure out which resource you want to try?
4. Do the current explanations as they stand make you “trust” these results, or is there anything you are unsure or unclear about?
   a. *Follow up:* After seeing these results, would you want more information? What would you want to know?
   b. *Follow up:* What is/are the most important piece of information that should be included? Why?
   c. *Follow up:* What is/are the least important pieces of information that are not necessary to include?
   d. *Follow up:* Do the current explanations help answer the questions you may have? What questions might you have about the portal? Are they clear?
5. Do these results bring about any feelings? Why?
   a. *Probe: For example, do these results make you feel: motivated, sad, discouraged, curious, confused?*
6. Is the FAQ page: Necessary? Helpful? Clear? Confusing?

*Once you fill out the 10-item questionnaire (after sign up) and the regular, bi-weekly questionnaire, a set of recommended resources will be displayed for you.*

Questions about the recommended resources displayed on the PoP portal:

1. What are the most important things that you would want to know about each resource that is recommended? Why?
2. Do you think that the results recommended here align with how you would describe your coping style?

*Optional (if time permits):*

1. Each recommended resource has a section that explains why it is being recommended for you as it relates to the pain coping profiles. Is this something that is helpful to include?
   a. *Follow up: Is the explanation easy to understand or not?*
   b. *Follow up: Is the explanation actually useful in helping you better understand why these resources are being recommended. If no - what kind of information are you looking for/expecting? If yes, please explain why.*
2. Each recommended resource also has another section that shares other, similar users’ interactions with the resources (whether they liked it or completed it). Would you like to see this information displayed, or not?
3. How do you feel about the level of detail that is displayed about each recommendation?
   a. *Probe: Is there too much or too little information?*

**General Comments:**

1. Would this new feature make users feel empowered when using the platform? “Empowered” can be defined as: make you feel confident in your judgment about whether a suggested resource is applicable to you or not.
   a. *Follow up: If yes, which features? Why? If no, why not?*
2. Would the new feature encourage users to use the platform? Why or why not?
3. Would the new feature make users feel engaged when using the portal? “Engaged” can be defined as whether you felt motivated, enabled, and/or invited to use it. Why or why not?

*Optional (if time permits):*

1. What did you like best from all the new features that were described today? Why?
2. Is there anything that you would want to change about the new features you saw today? Why?
3. Is there anything that we did not cover today that you would like to share?
4. Do you have any questions before we conclude the interview?

**Interview Ending**

*Thank you for taking the time to participate in the interview.*

Table 1. Alignment of perceived-value dimensions with representative interview questions

| Dimension | Description | Representative interview questions |
| --- | --- | --- |
| Framing | The extent to which the questions, response options, and results are presented in a way that feels supportive, engaging, and aligned with users’ needs and expectations. | “Did these questions bring about any feelings?” “Did these questions make you think about anything specifically?” |
| Clarity | The ease and effort with which users can read, interpret, and understand the questions, response options, results, and supporting explanations. | “Are the questions written in a way that is easy for you to understand?” “Is there anything about the results that you find confusing?” |
| Informativeness | The extent to which the questionnaire, results, and resource descriptions provide sufficient information to support interpretation. | “Do the current explanations as they stand make you ‘trust’ these results, or is there anything you are unsure or unclear about?” “After seeing these results, would you want more information? What would you want to know?” |
| Applicability | The extent to which the questionnaire, results, and recommendations feel relevant and personally meaningful to users’ experiences, circumstances, and needs. | “Are all the questions relevant to helping you find the best approach in managing your chronic pain?” “How do the results from the questionnaire compare to how you cope with pain?” |
| Usefulness | The extent to which users perceive the questionnaire, results, and recommendations as beneficial in guiding decisions and actions. | “Do you feel that this feature is useful for you? Why or why not?” “Would this questionnaire help you to pick resources from the PoP portal?” |
