# Supplementary material for: Impact of coping profiles on social identity in a youth chronic pain recommender system: qualitative descriptive study
Source: Front Digit Health. 2026 Sep 15;8:1727940. doi: 10.3389/fdgth.2026.1727940 (PMC13619948; doi:10.3389/fdgth.2026.1727940)
Supplement: Supplementary file 3 [file Supplementaryfile3.docx]

# Supplementary file 3: Consolidated criteria for reporting qualitative research (COREQ) Guidelines

*Table 1: Reporting items from the CORE-Q Guidelines, with associated page numbers and comments, as applicable.*

| **No Item** | **Guide questions/description** | **Location in paper and/or Comments** |
| --- | --- | --- |
| **Domain 1: Research team and reflexivity - Personal Characteristics** | | |
| 1. Interviewer/facilitator | Which author/s conducted the interview or focus group? | Methods: Overview |
| 2. Credential | What were the researcher’s credentials? E.g., PhD, MD | Additional file 4 |
| 3. Occupation | What was their occupation at the time of the study? | Additional file 4 |
| 4. Gender | Was the researcher male or female? | Additional file 4 |
| 5. Experience and training | What experience or training did the researcher have? | Additional file 4 |
| **Domain 1: Research team and reflexivity - Relationship with participants** | | |
| 6. Relationship established | Was a relationship established prior to study commencement? | AN attended the interviews and had prior relationships. |
| 7. Participant knowledge of interviewer | What did the participants know about the researcher? e.g. personal goals, reasons for doing the research? | Additional file 2 (interview guide) |
| 8. Interviewer characteristics | What characteristics were reported about the interviewer/facilitator? e.g. Bias, assumptions, reasons and interests in the research topic | Additional file 2 (interview guide) |
| **Domain 2: Study design - Theoretical framework** | | |
| 9. Methodological orientation and Theory | What methodological orientation was stated to underpin the study? e.g. grounded theory, discourse analysis, ethnography, phenomenology, content analysis | Methods: Overview |
| **Domain 2: Study design - Participant selection** | | |
| 10. Sampling | How were participants selected? e.g., purposive, convenience, consecutive, snowball | Methods: Participant Selection and Characteristics |
| 11. Method of approach | How were participants approached? e.g., face-to-face, telephone, mail, email | Methods: Overview and Participant Selection and Characteristics |
| 12. Sample size | How many participants were in the study? | Methods: Participant Selection and Characteristics |
| 13. Non-participation Setting | How many people refused to participate or dropped out? Reasons? | No participants dropped out. |
| 14. Setting of data collection | Where was the data collected? e.g., home, clinic, workplace | Methods: Data Collection and Analysis |
| 15. Presence of nonparticipants | Was anyone else present besides the participants and researchers? | No |
| 16. Description of sample | What are the important characteristics of the sample? e.g. demographic data, date | Methods: Participant Selection and Characteristics |
| **Domain 2: Study design - Data collection** | | |
| 17. Interview guide | Were questions, prompts, and guides provided by the authors? Was it pilot tested? | Methods: Data Collection and Analysis; Additional file 2 |
| 18. Repeat interviews | Were repeat interviews carried out? If yes, how many? | No |
| 19. Audio/visual recording | Did the research use audio or visual recording to collect the data? | Methods: Data Collection and Analysis |
| 20. Field notes | Were field notes made during and/or after the interview or focus group? | Methods: Data Collection and Analysis |
| 21. Duration | What was the duration of the interviews or focus group? | Methods: Overview |
| 22. Data saturation | Was data saturation discussed? | Methods: Data Collection and Analysis |
| 23. Transcripts returned | Were transcripts returned to participants for comment and/or correction? | No, but an effort was made during the interviews to clarify and confirm feedback. |
| **Domain 3: Analysis and Findings - Data Analysis** | | |
| 24. Number of data coders | How many data coders coded the data? | Methods: Data Collection and Analysis |
| 25. Description of the coding tree | Did the authors provide a description of the coding tree? | Methods: Data Collection and Analysis |
| 26. Derivation of themes | Were themes identified in advance or derived from the data? | Methods: Data Collection and Analysis |
| 27. Software | What software, if applicable, was used to manage the data? | Methods: Data Collection and Analysis |
| 28. Participant checking | Did participants provide feedback on the findings? | No, but an effort was made during the interviews to clarify and confirm feedback. |
| **Domain 3: Analysis and Findings - Reporting** | | |
| 29. Quotations presented | Were participant quotations presented to illustrate the themes/findings? Was each quotation identified? e.g., participant number | Results |
| 30. Data and findings consistent | Was there consistency between the data presented and the findings? | Results |
| 31. Clarity of major themes | Were major themes clearly presented in the findings? | Results |
| 32. Clarity of minor themes | Is there a description of diverse cases or a discussion of minor themes? | No minor themes identified. |
