# Supplementary material for: Impact of coping profiles on social identity in a youth chronic pain recommender system: qualitative descriptive study
Source: Front Digit Health. 2026 Sep 15;8:1727940. doi: 10.3389/fdgth.2026.1727940 (PMC13619948; doi:10.3389/fdgth.2026.1727940)
Supplement: Supplementary file 4 [file Supplementaryfile4.docx]

# Supplementary file 4: Positionality Statements

Author AB is a second-generation Canadian settler who identifies as a white, cisgender woman from a middle-class background. She is a biomedical engineer and health services research trainee, currently pursuing her doctoral degree at the University of Toronto. AB has extensive experience in developing digital health software and has been actively involved in evaluating the performance and acceptability of these emerging health technologies. While AB has lived experience as a caregiver within her family, she has had no prior experience or direct interaction with individuals diagnosed with chronic pain. As such, she acknowledges the limitations in her ability to fully comprehend the lived experiences of these individuals. Drawing on her research training and personal caregiving experiences, AB conducted the interviews and completed the data analysis.

Author JS is a second-generation Canadian settler who identifies as a middle eastern, cis-gender male from a middle-class background. He is currently pursuing a masters in Health Systems Research (Health Informatics emphasis) at the University of Toronto. JS did not complete interviews during the data collection phase, and is only familiar with study participants through the transcripts of participant interviews.

Author AN is the research coordinator for the study conducted at The Hospital for Sick Children (SickKids). She is a brown, cisgender woman, who is responsible for study administration and participant recruitment. She recruited and consented each participant who completed the interview. She sat in on the interviews to monitor and ensure participant safety and to answer any study queries. AN dealt with participant compensation and did not directly interview any of the participants.
